# ASSOCIATION BETWEEN WITHIN-DAY CALORIE INTAKE PATTERNS AND MORTALITY RISK AMONG US ADULTS

**DOI:** 10.1093/geroni/igae098.2397

**Published:** 2024-12-31

**Authors:** Ziling Mao, Anne Newman, Samaneh Farsijani

**Affiliations:** University of Pittsburgh, Pittsburgh, Pennsylvania, United States; University of Pittsburgh, University of Pittsburgh, Pennsylvania, United States; University of Pittsburgh, Pittsburgh, Pennsylvania, United States

## Abstract

Nutritional research on longevity has historically prioritized quantity of intake, with low-calorie diets as a prime example. Yet the crucial effects of intake timing and distribution on circadian rhythm and health are largely unexplored. Here, we determined the association between 24-hour calorie intake patterns and all-cause mortality.

**Methods:**

We included 33,094 nationally representative US adults (47.6±0.2y; 52.4% women) from NHANES. First, we calculated calorie intake at 6-hour intervals within a day via 24-hour food recalls. Then, group-based trajectory analysis was performed, and four distinct temporal patterns of daily calorie intake were identified: “Skewed-to-Morning”, “Skewed-to-Midday”, “Skewed-to-Evening”, and “Midday-Evening-Balance”. Mortality was ascertained through December 2019 using the National Death Index. Survey-weighted Cox hazard regression was used to determine the association between calorie intake patterns and all-cause mortality, adjusting for demographics, income, BMI, physical activity, smoking, alcohol, calorie intake, and diet quality.

**Results:**

Among participants, 18% had morning-skewed, 22% midday-skewed, 22% evening-skewed calorie intake, and 38% had equal intake between midday and evening. During a median follow-up of 96 months, 4,162 participants died. In the unadjusted model, all skewed calorie intake patterns were associated with higher all-cause mortality compared to the Midday-Evening-Balance group (P< 0.05). After multivariable adjustment, Skewed-to-Morning (HR=1.18; 95%CI, 1.04-1.35) and Skewed-to-Evening (HR=1.20; 1.02-1.39) patterns remained significantly associated with higher mortality.

**Conclusion:**

Calorie intake patterns skewed toward morning or evening may be associated with a higher mortality risk, independent of amount and quality of diet. This underscores the importance of considering both the quantity and the timing of daily calorie consumption.

